# Intestinal absorbability of three *Radix Puerariae *isoflavones including daidzein, daidzin and puerarin

**DOI:** 10.1186/1749-8546-6-41

**Published:** 2011-11-23

**Authors:** Li Zhang, Antony Kin Pan Siu, Ge Lin, Zhong Zuo

**Affiliations:** 1 School of Pharmacy, Faculty of Medicine, The Chinese University of Hong Kong, Shatin, New Territories, Hong Kong SAR, China; 2 School of Biomedical Sciences, Faculty of Medicine, The Chinese University of Hong Kong, Shatin, New Territories, Hong Kong SAR, China

## Abstract

**Background:**

*Radix Puerariae *(*Gegen*) contains abundant isoflavones in the forms of glycosides and aglycones, such as daidzein, daidzin and puerarin. This study aims to investigate the intestinal absorbability and mechanism of these three structurally related isoflavones.

**Methods:**

The bi-directional transport of these three isoflavones in Caco-2 monolayer model was performed to evaluate their absorbability and involvement of transporters in Transwell. *In vitro *incubation of daidzin and puerarin with rat intestinal microvilli preparation was conducted to estimate their potential form of absorption *in vivo*.

**Results:**

Daidzein demonstrated passive diffusion transport while puerarin did not. Daidzin showed basolateral-to-apical transport and the absorption extent could be reduced by 50% in the presence of MK571, a multidrug resistance-associated protein inhibitor (MRP). The *in vitro *incubation study of daidzin and puerarin indicated that daidzin was hydrolyzed to daidzein whereas puerarin remained unchanged.

**Conclusion:**

While daidzein was transported more efficiently, puerarin was resistant to intestinal hydrolysis and inefficiently transported across intestinal epithelium. Daidzin demonstrated a low intestinal absorbability due to a significant efflux transport mediated by MRPs. Daidzin was likely to be hydrolyzed by intestinal microvilli and subsequently released daidzein for intestinal absorption.

## Background

**Figure 1:**
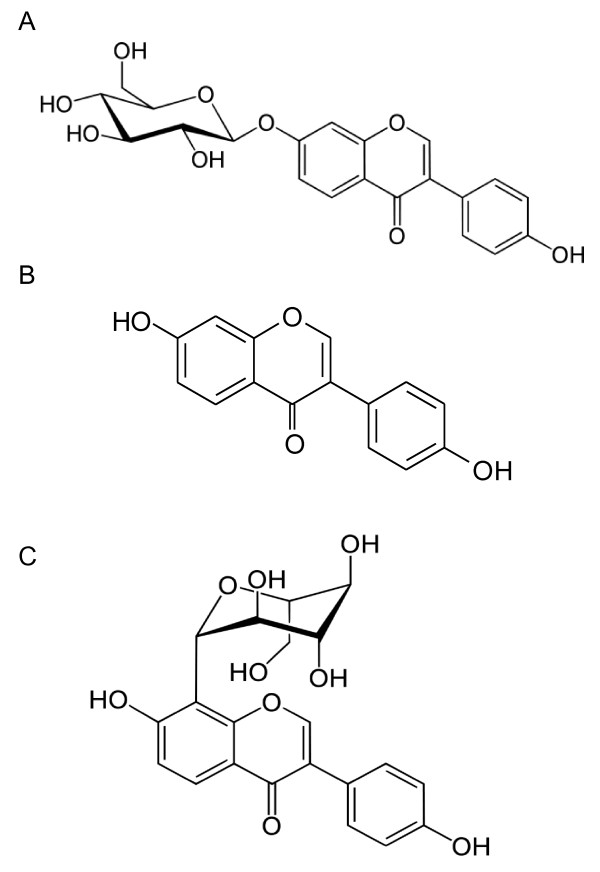
**Chemical structures of the three selected isoflavones**. A Daidzin; B Daidzein; C Puerarin.

*Radix Puerariae*, a Chinese medicinal plant, contains three major isoflavones, namely daidzein, daidzin and puerarin (Figure 1). Daidzin, which was reported to inhibit aldehyde dehydrogenase-2 (ALDH-2) in isolated mitochondria, may be useful as an anti-alcohol agent [1,2]. Puerarin is used to relieve thirst and treat common cold and neck stiffness due to hypertension [3].

Despite various pharmacological activities reported for isoflavones, only a few pharmacokinetics studies on the soybean isoflavones were reported [4,5]. Izumi *et al*. found that the absorption rate of aglycones were higher than their corresponding glycosides due to their higher lipophilicities and smaller molecular sizes [5]. Setchell *et al*. indicated that isoflavone glycosides such as daidzin and genistin in the blood sample were barely detectable whereas a large amount of isoflavone aglycone and its conjugated glucuronides were found in blood [6]. Another study suggested that the *in vivo *bioavailability of isoflavone glycosides could be enhanced by increasing the activities of beneficial intestinal bacteria which were capable of hydrolyzing the glycosides to their corresponding aglycones [7]. Moreover, isoflavones were reported to undergo enterohepatic recirculation [8,9], suggesting that certain transport mechanisms other than passive diffusion may involve in the absorption of isoflavones such as multi-resistance protein (MRP) transporters [10,11]. On the other hand, puerarin may exhibit different pharmacokinetics because the intestinal β-glucosidase is reported to be weakly effective in catalyzing the hydrolysis of puerarin like C-glucoside [12]. Yasuda *et al*. [12] showed that puerarin was detected in the blood and that conjugated metabolites of both puerarin and daidzein were found in urine, suggesting that puerarin could be absorbed in its intact form while part of it could be hydrolyzed to daidzein before being absorbed in the gastrointestinal tract. Kim *et al*. demonstrated the intestinal bacterial mediated biotransformation of puerarin to daidzein [13].

Previous findings focused on the intestinal bacterial hydrolysis during the absorption of isoflavone glycosides. For *in vivo *studies, Caco-2 cell monolayer model [14] was chosen for the investigation of the absorption mechanisms of the three isoflavones and the comparison of absorption between the glycoside and its aglycone in a controlled environment. This study aims to investigate the intestinal absorption mechanisms of daidzin, daidzein and puerarin by using human Caco-2 cell monolayer model as well as *in vitro *rat microvillus hydrolysis model.

## Methods

### Chemical and reagents

Daidzin was purchased from Fluka Chemie (Germany). Puerarin and daidzein were provided by DH Chen from the Institute of Medicinal Plant Development, Chinese Academy of Medical Sciences, China. Analytical-graded acetonitrile (ACN) were purchased from Riedel-de Haën (Germany). Sodium dihydrogenphosphate dihydrate was purchased from BDH Chemical (UK). Analytical grade dimethyl sulphoxide (DMSO) was from Lab-Scan Analytical Sciences (Thailand). Phosphate buffered saline tablets were purchased from Sigma Chem Co (USA). MK571 was supplied by Biomol Research Laboratory (USA).

Caco-2 human colon carcinoma cell line was obtained from American Type Culture Collection (ATCC number HTB37, USA). Dulbecco's Modified Eagle's Medium, fetal bovine serum, nonessential amino acid, L-glutamine and trypsin-EDTA used in cell culture were purchased from GibcoBRM (USA). Transwell^® ^inserts (sterile Polycarbonate filter) were purchased from Corning Costar Corporation (USA).

### Physicochemical characteristics of the selected isoflavones

#### Stability of the selected isoflavones in transport buffer

The transport buffer (PBS^+^) used in the Caco-2 monolayer model was prepared as previously described [15]. Briefly, 20 μg/ml of puerarin and daidzin and 0.5 μg/ml of daidzein solutions in PBS^+ ^at pH6.8 or pH7.4 were kept in 37°C water bath. For stability tests at 37°C, samples were taken at 0, 15, 40, 75, 120 and 180 minutes. At each time interval, 300 μl of isoflavone solution was taken and then acidified with 50 μl of 0.425% phosphoric acid. All experiments were conducted in triplicates. The amount of selected isoflavones remained in PBS^+ ^was determined with high-performance liquid chromatography (HPLC).

#### Partition coefficients of the selected isoflavones

The aqueous solution (10 ml) containing individual isoflavones was mixed with octanol (3 ml). The mixtures were equilibrated in tube rotator for 24 hours followed by sampling from the aqueous layers and determining concentrations of the isoflavones by HPLC. The test was performed in triplicates for each isoflavone. The partition coefficients (*D*) at both pH7.4 and pH 6.8 were obtained through the following equation:

$$Log\;D=log\;\left({\left[\text{isoflavone}\right]}_{\text{octanol}\;\text{phase}}∕{\left[\text{isoflavone}\right]}_{\text{aqueous}\;\text{phase}}\right)$$

### Bidirectional transport studies of the selected isoflavones in the Caco-2 monolayer model

#### Caco-2 cell culture

The Caco-2 cells were cultured and seeded in the Transwell^® ^inserts (24 mm, 0.4 μm pore size, 4.71 cm^2^, polycarbonate filter) according to our previous study [15]. Cells used for the current study were from passage 35-40. The transport experiments were carried out at 23-25 days after seeding.

#### Apical-to-basolateral (A→B) transport studies

Upon removal of culture medium, the Transwells^® ^were pre-equilibrated with PBS^+ ^at 37°C for 15 minutes. Then 1.5 ml of the isoflavone solution was loaded to the apical (donor) chamber of the Transwell^® ^while 2.6 ml of blank PBS^+ ^was loaded to the basolateral (receiver) chamber. For the transport study of daidzein, 0.5 ml of the sample was taken from the receiver side at certain time interval (15, 30, 45, 60 and 90 minutes) followed by replacing with an equal volume of blank PBS^+^. Samples were also obtained from the donor side at 90 minutes. For the transport studies of puerarin and daidzin, samples were taken at 30, 60, 90, 120, 150 and 180 minutes from receiver side and at 180 minutes from the donor side. All Caco-2 transport studies were performed at least in triplicates.

#### Basolateral-to-apical (B→A) transport studies

Procedure was similar to the A→B transport studies except for the donor side and the receiver side was reversed. Loading solution (2.6 ml) was added into the basolateral side and 1.5 ml of the blank PBS^+ ^was added to the apical side. Samples were taken from the receiver (apical) side at the same time points and at the end of the experiment from the donor (basolateral) side as mentioned for the A→B studies. The studies were also performed at least in triplicates.

#### Basolateral-to-apical transport studies of the selected isoflavones in the presence of MK571

The study was performed only for isoflavones that had shown a favorable B→A transport in the bi-directional transport studies. As demonstrated in our previous study [15], MK571 is a MRP inhibitor. To investigate the influence of MRP on the efflux transport of the selected isoflavones, we preloaded 1.5 ml of MK571 (50 μM) at apical side and 2.6 ml of MK571 (50 μM) at the basolateral side respectively. The B→A transport experiments of the selected isoflavones in the presence of MK571 (50 μM) in the both apical and basolateral chambers were then performed following the method described above. The studies were performed in triplicates.

### Potential hydrolysis of daidzein glycosides during intestinal absorptions

Male Sprague-Dawley rats, supplied by and bred at the Laboratory Animal Service Center at The Chinese University of Hong Kong, were housed in an air-conditioned room under a 12/12-hour light/dark cycle. The experiment was conducted after being approved by the Animal Ethics Committee of The Chinese University of Hong Kong. The rat intestinal microvilli preparations (protein content: 0.43 mg/ml) were collected as previously described [16] and incubated with daidzin and puerarin (50 μM) at 37°C for two hours before we added the same volume of ice-cold methanol to terminate the reaction.

### Sample treatment and analysis

Samples obtained in the Caco-2 transport studies were acidified with 0.425% phosphoric acid at a sample to acid ratio of 150:25 (v/v). An aliquot of acidified samples (100 μl) was injected into the HPLC for the measurement of the isoflavone concentrations. Waters 2695 separation modules and Waters 996 photodiode array detector were used in the HPLC analyses of the Caco-2 samples. A reversed-phase column (100 × 4.6 mm 5μ Apex ODS Symm, Jones Chromatography, USA) equipped with a guard column (7.5 × 4.6 mm 5μ Spherisorb^® ^ODS-2 C_18_, Waters, USA]) was used for the analyses of all three isoflavones. In order to shorten sample-running time, three separated HPLC methods were used for each isoflavone.

For the analysis of daidzein, an isocratic mobile phase of 27% ACN in 0.025 M phosphate buffer (pH2.5) at a flow rate of 1 ml/min was used for the analysis of daidzein. For the analysis of puerarin, a gradient mobile phase consisting of ACN and 0.025 M phosphate buffer (pH2.5) was used. The gradient program started with a 2-minute run of 10% ACN and 90% of phosphate buffer, then the percentage of ACN was increase to 15% in 10 minutes followed by a decrease of 10% in the following three minutes and maintaining the percentage for another five minutes. Similar to puerarin, a gradient mobile phase containing ACN and 0.025 M phosphate buffer (pH2.5) was used for the detection of daidzin. The elution started with 18% ACN and 82% buffer, followed by an increase of the proportion of ACN to 30% in the next two minutes and then maintaining for three minutes before returning to the initial condition in two minutes and remaining with the same elution condition for another four minutes. Daidzein was detected at 300 nm whereas daidzin and puerarin were detected at 250 nm. The detection limits of the selected isoflavones were the lowest concentration of the compounds that could produce peak height three times greater than that of the background noise. The concentrations of prepared isoflavone standard solutions ranged from 0.0125-1 μg/ml.

Samples from the *in vitro *incubation studies were centrifuged at 16,000 × *g *(Eppendorf, Germany) for five minutes; 50 μl of the supernatant of the samples were obtained and injected into the HPLC for analysis. In order to monitor daidzin, puerarin and daidzein in a single run for the samples from the hydrolysis study, we modified the above HPLC method for simultaneous detection of the three compounds. The analytes were separated with an HPLC column (BDS reversed phase column, 25 cm × 4.6 mm id; 5 μm particle size, Thermo Hypersil, UK) and eluted by the linear gradient elution at a flow rate of 1 ml/min. The gradient started with 95% of water with 0.01% aqueous acetic acid (A) and 5% of ACN containing 0.01% acetic acid (B), changed linearly to 83% A and 17% B in 30 minutes; 70% A and 30% B in 30-40 minutes; 50% A and 50% B in 40-45 minutes; and then back to the initial composition in 15 minutes followed by ten minutes after equilibrium.

### Data analysis

The apparent permeability coefficient, *P_app _*(cm/s), was calculated as follows [14].

$$P_{app}=\frac{dC∕dT\times V}{\left(A\times C_{o}\right)}$$

where *dC/dT *is the initial slope of the plot of cumulative isoflavone concentrations versus time; *V *is the volume of receiver chamber (ml); *A *is the apparent surface area of the monolayer; *C_o _*is the initial concentration in donor site (μmol/ml). In addition to *P_app_*, the percentage of recovery was calculated for each transport study to determine the extent of absence of isoflavone, which may be due to cell uptake or metabolism of the selected isoflavones after passing through the Caco-2 cells. It was calculated as follows.

$$\begin{array}{l} \text{Recovery}\%=\text{Final amount }\left(\text{in }\mu \text{mole}\right)\text{ of isoflavone in donor and receptor side}∕\text{Initial }\\ \text{amount }\left(\text{in }\mu \text{mole}\right)\text{ of isoflavone in donor side} \end{array}$$

The reported values are expressed as mean ± standard deviation (SD). Unpaired Student's *t*-test was used to evaluate the statistically significance of difference between two groups. A value of *P *< 0.05 was considered statistically significant for all tests.

## Results

### HPLC analysis

The calibration curves of daidzein, puerarin and daidzin were linear throughout the studied concentration range with correlation coefficients greater than 0.999. The detection limits of these three isoflavones were 50 ng/ml for daidzein, 25 ng/ml for daidzin and 12.5 ng/ml for puerarin. Inter-day verification was conducted with three sets of calibration curve determined at different days. Intra-day verification was performed on three sets of data measured on the same day. Analyses of all isoflavones showed percentage deviation of less than 10% for all concentrations tested.

### Physicochemical characteristics of the selected isoflavones

After three hours of incubation at pH6.8/pH7.4, 95.50 ± 0.84%/97.73 ± 3.43%, 105.00 ± 2.72%/106.74 ± 4.20% and 108.66 ± 2.16%/108.11 ± 5.21% of daidzein, puerarin and daidzin remained respectively, indicating that the selected isoflavones were considerably stable in PBS^+ ^under all pH conditions and suitable for the Caco-2 transport studies. To mimic the physiological pH of human intestine, we chose pH6.8 for the transport studies.

The *D *of the isoflavones at pH7.4 was 0.06 ± 0.02 for puerarin, 0.80 ± 0.03 for daidzin and at least 2.58 ± 0.02 for daidzein which corresponded to the order of their lipophilicities, *ie *daidzein > daidzin > puerarin. The *D *at pH 6.8 was also measured which was greater than 2.58 ± 0.02, 0.34 ± 0.03, -0.42 ± 0.15 for daidzein, daidzin, puerarin respectively. The order of lipophilicities of the studied isoflavones at the two studied pH conditions was the same.

### Bi-directional transport studies of isoflavone solutions in the Caco-2 monolayer model

The loading concentrations for the three isoflavones solutions in Caco-2 transport studies were 18.25 ± 0.11 μg/ml for daidzein, 22.10 ± 1.11 μg/ml for puerarin and 18.55 ± 0.34 μg/ml for daidzin. The results of bi-directional transport studies of the three selected isoflavones are summarized in Table 1. Daidzein was detectable in receiver chambers 15 minutes after being loaded with similar *P_app _*and percentage recoveries for both directional transport studies. On the other hand, puerarin did not demonstrate passive diffusion either in 120 minutes after apical loading or 60 minutes after basolateral loading in the Caco-2 monolayer model. The cumulative concentrations of puerarin found at 180 minutes in the receiver sides was 0.043 ± 0.006 μM after apical loading and 0.12 ± 0.02 μM after basal loading with the percentage of recoveries above 89%. In summary, from both directional transport studies, puerarin was barely detectable in the receiver sides (only around 0.3% of puerarin permeable through the Caco-2 monolayer). Daidzin showed a significant difference (t = 24.5, *P *< 0.0001) between its two directional transport studies with undetectable daidzin in receiver chamber from all sampling times after apical loading and a more efficient transport after basolateral loading. Therefore, carrier mediated transport of daidzin from B→A side was suggested and the inhibition transport study on daidzin using MK571 was performed afterwards.

**Table 1:** Bi-directional transport of the selected isoflavones in Caco-2 monolayer model

Isoflavones	Apical to basolateral		Basolateral to apical	
	
	***P***_***app ***_**(cm/s)**	Recovery (%)	***P***_***app ***_**(cm/s)**	Recovery (%)
Daidzein	34.2 ± 7.06 × 10^-6^	83.12 ± 5.74	38.6 ± 1.81 × 10^-6^	83.96 ± 14.1
Puerarin	ND	88.91 ± 7.03	ND	92.73 ± 8.09
Daidzin	ND	92.94 ± 1.77	5.30 ± 0.20 × 10^-7^	99.17 ± 2.55

The data are expressed as mean ± SD (*n *= 3). ND: not detectable

### B→A transport study of daidzin in the presence of MK571

**Figure 2:**
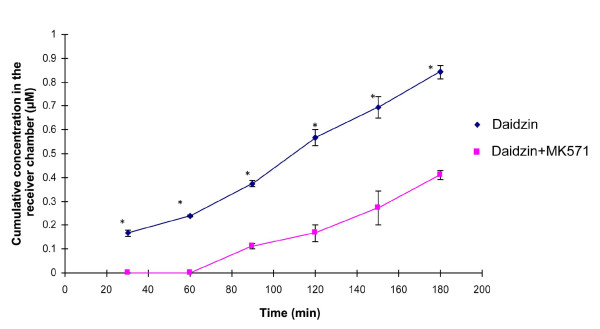
**Basolateral-to-apical transport of daidzin in the absence and presence of MK 571, the MRP inhibitors**.

Figure 2 shows the cumulative concentration versus time profiles for the basolateral-to-apical transport of daidzin in the absence and presence of MK571. In the presence of MK 571, daidzin was only detectable in the receiver chamber starting from 90 minutes after being loaded. The cumulative concentration of daidzin in the receiver chamber was 0.41 ± 0.02 μM at 180 minutes, which was about 50% lower than that without MK571. The percentages of recovery of daidzin for these transport studies were all above 90%.

### Hydrolysis of daidzin and puerarin during intestinal absorptions

**Figure 3:**
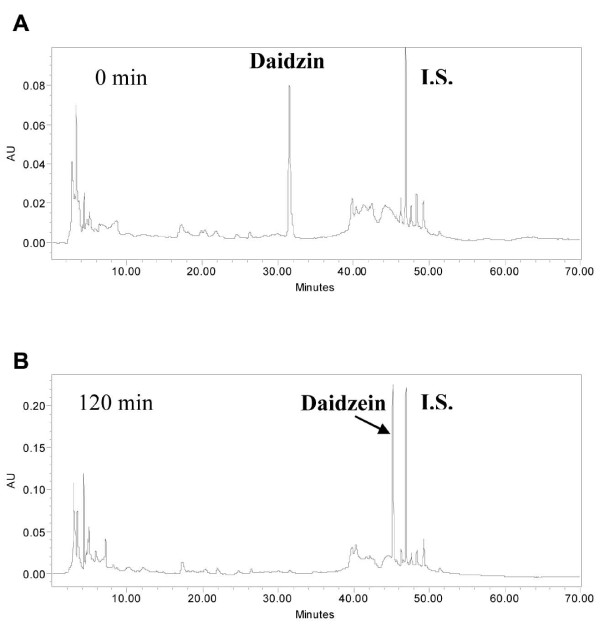
**The HPLC-UV chromatograms of daidzin before and after incubations with rat intestinal microvilli preparations**. I.S.: internal standard.

**Figure 4:**
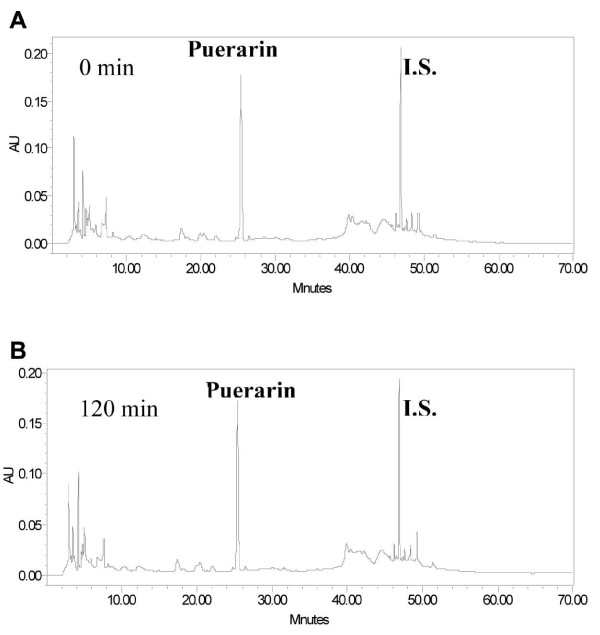
**The HPLC-UV chromatograms of puerarin before and after incubations with rat intestinal microvilli preparations**. I.S.: internal standard.

As shown in Figures 3 and 4, after incubation with rat intestinal microvilli preparations for two hours, the majority of puerarin remained unchanged (Figure 4) whereas daidzin (Figure 3) were completely hydrolyzed to daidzein with no detectable daidzin in the incubation medium.

## Discussion

Daidzein, puerarin, and daidzin, the three selected isoflavones for this study, have a common isoflavone nucleus and structurally exist as aglycone, C-glucoside and O-glucoside respectively. The results of this study show that these three compounds transported differently across the Caco-2 cell monolayer, thereby exhibiting different transport mechanism when absorbed through the human gastrointestinal tract. The physicochemical tests performed on the three compounds provided information on the properties of the selected isoflavones, which possibly affected their transport through the Caco-2 cell monolayer.

As indicated by the rankings of *D *at both pH6.8 and pH7.4, there was a large difference (over 200-fold, *P *< 0.0001) in the *D *between isoflavone aglycone and its glycosides. Daidzein was the most lipid-soluble one with the concentration of daidzein barely detectable in the aqueous layer. Therefore, the *log P *and *log D *values of daidzein were estimated based on the HPLC detection limit of it. Daidzin demonstrated a moderate *log P *value with a slight preference in octanol. Puerarin had the lowest *log P *value among the three, suggesting its preference in water.

The bi-directional transport studies of the three selected isoflavones in the Caco-2 monolayer model found that the *P_app _*values of daidzein were the highest, with both *P_app A to B _*and *P*_*app B to A *_greater than 10^-5^cm/s. Moreover, the *P_app _*value of daidzein found in both directional transport studies were similar, indicating that the compound was likely to be mainly transported across the monolayer by passive diffusion. The percentage of recovery for daidzein after transport studies was about 83% for both directional transport studies, which was lower than that obtained from puerarin and daidzin. Since the three isoflavones were all confirmed to be stable in PBS^+ ^under the experimental condition, the cell uptake and intracellular metabolism of daidzein may contribute to its relatively lower recovery, which was also suggested by Murota *et al*. [17].

In this study, puerarin was poorly transported through the Caco-2 cell monolayer with its concentration in receiver chamber not being detectable until two hours. Only about 0.3% of puerarin passed through the monolayer in directional transport studies, indicating that the two directional permeabilities of puerarin were similar and puerarin likely passed through the monolayer with the passive diffusion pathway. Assuming that puerarin was mainly absorbed in the gut by passive diffusion, we think the role of intestinal bacteria in breaking down it into daidzein may be important. However, no hydrolysis of puerarin was shown by Yasuda *et al*. [12] and this study. The absorption mechanism of puerarin was probably different from that of the other studied isoflavones. This study only established the preliminary absorption profile of puerarin, further investigations are needed to study the relationship between absorbed puerarin and its pharmacological effects.

Daidzin had a different transport profile from the other two isoflavones with a significant efflux medicated by MRP, which was consistent with Vaidyanthan *et al *[11]. As multidrug resistance-associated protein 2 (MRR2) was responsible to transport various compounds containing O-glucuronides including estradiol 17β-D-glucuronide [18], it was also possible to mediate the efflux transport of daidzin, due to their structural similarity.

Two factors may contribute to the absorption differences between daidzein and daidzin. Izumi *et al*. [5] suggested that daidzin is a more hydrophilic and bigger molecular in molecular size which may affect its absorption through passive diffusion pathway. Another factor was the efflux of daidzin, which would prevent it from being transported across the intestinal wall. These two factors may explain the different findings in the absorption of daidzein and daidzin. On the other hand, as active transport mechanisms could not be found in the transport of puerarin, the limited absorption of puerarin was likely due to its large molecular size and high hydrophilicity. Between the absorptions of daidzin and puerarin, daidzin showed a significant B→A efflux whereas puerarin did not, indicating that puerarin was unlikely to be the substrate of MRP transporters.

The incubation with rat intestinal microvilli preparations demonstrated that daidzin could release its aglycone daidzein while puerarin was relatively stable during their absorption processes in. Due to the high polarities and poor A→B permeabilities of daidzin and puerarin, it was not likely that these two glycosides of daidzein could be absorbed directly at small intestine *in vivo*. However, daidzin could be absorbed after hydrolysis at small intestine to daidzein which could be absorbed due to its high lipophilicity.

## Conclusion

While daidzein was transported more efficiently, puerarin was resistant to intestinal hydrolysis and inefficiently transported across intestinal epithelium. Daidzin demonstrated a low intestinal absorbability due to a significant efflux transport mediated by MRPs. Daidzin was likely to be hydrolyzed by intestinal microvilli and subsequently released daidzein for intestinal absorption.

## Competing interests

The authors declare that they have no competing interests.

## Authors' contributions

GL and ZZ designed the study. LZ and AKPS carried out the experiments. LZ, AKPS and ZZ: performed the data analysis. All authors have read and approved the final manuscript.
